# Effects of early local administration of high-dose bFGF on a recurrent laryngeal nerve injury model

**DOI:** 10.1186/s40463-023-00647-4

**Published:** 2023-07-24

**Authors:** Takao Goto, Rumi Ueha, Taku Sato, Tatsuya Yamasoba

**Affiliations:** 1 Department of Otorhinolaryngology and Head and Neck Surgery, Faculty of Medicine, The University of Tokyo, 7-3-1 Hongo, Bunkyo-Ku, Tokyo, 113-8655 Japan; 2 The University of Tokyo Hospital Swallowing Center, Tokyo, Japan; 3 Tokyo Teishin Hospital, Tokyo, Japan

**Keywords:** Basic fibroblast growth factor, Vocal fold paralysis, Regeneration, Muscle satellite cells, Neuromuscular junction

## Abstract

**Background:**

Research on regenerative medicine using basic fibroblast growth factor (bFGF) has recently advanced in the field of laryngology. We previously reported that local administration of bFGF 1 month after recurrent laryngeal nerve (RLN) paralysis compensated for atrophy of the thyroarytenoid muscle. The objective of this study was to elucidate the effects of early bFGF administration on the thyroarytenoid muscle after RLN transection and to investigate the underlying mechanisms.

**Methods:**

A rat model of RLN paralysis was established in this study. One day after RLN transection, low- (200 ng) or high-dose (2000 ng) bFGF or saline (control) was administered to the thyroarytenoid muscle. The larynges were excised for histological and immunohistochemical examinations at 1, 7, 14, 28, and 56 days after administration.

**Results:**

The cross-sectional thyroarytenoid muscle area was significantly larger in the high-dose group than in the saline and low-dose groups on days 28 and 56. Immunohistochemistry indicated that bFGF significantly increased the number of satellite cells in the thyroarytenoid muscle up to day 14 and that of neuromuscular junctions on days 28 and 56.

**Conclusions:**

A single, early local administration of high-dose bFGF prevented atrophic changes in the thyroarytenoid muscles by activating satellite cell proliferation and reforming neuromuscular junctions. As increased neuromuscular junctions are expected to maintain myofiber volume, bFGF administration may prevent thyroarytenoid muscle atrophy in the mid to long term.

**Graphical abstract:**

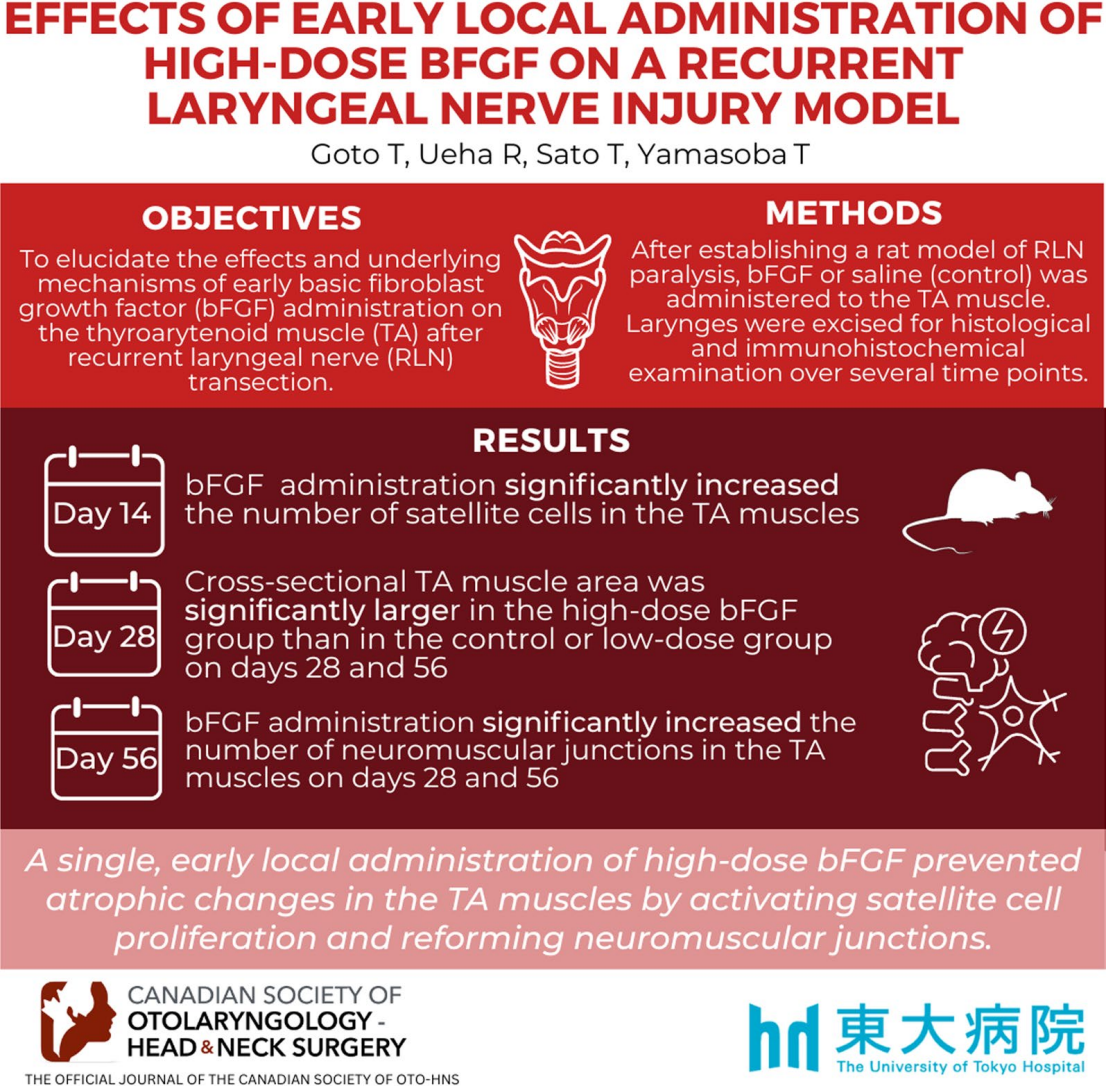

## Background

The voice is an important tool for communication and is produced mainly by the vibration of the vocal folds. Unilateral vocal fold paralysis (UVFP) is caused by recurrent laryngeal nerve (RLN) dysfunction, including vagal nerve disorders. It usually weakens the tension of the affected vocal fold, lessens the volume of the paralyzed vocal fold, and causes the phonatory function to decline.

For hoarseness due to UVFP, surgical treatments, such as local injection of various materials into the paralyzed vocal fold, including collagen [1] or autologous fat [2], and laryngeal framework surgery [3] are commonly performed. These treatments provide only static adjustment to the larynx, and their effects may be limited by the remaining hoarseness due to progressive atrophy of the paralyzed thyroarytenoid (TA) muscle. Therefore, medications that can prevent the progression of muscle atrophy or promote muscle regeneration are in high demand. In the human larynx, several anastomoses exist between the laryngeal nerves, such as Galen’s anastomosis and the arytenoid plexus [4]. These anastomoses include nerve connections between the superior laryngeal nerve (SLN) and RLN in the larynx. Considering that reinnervation of the TA muscle can occur even after UVFP through these anastomoses, it would be ideal if drugs that promote nerve regeneration could be used to treat UVFP.

Satellite cells (SCs), which are precursors of skeletal muscles, such as the TA muscle, play a key role in muscle homeostasis and regeneration. SCs　are normally in a quiescent state in adult muscles, and start proliferating during muscle regeneration in response to denervation. After several rounds of proliferation, most SCs differentiate into myoblasts, which fuse to form new myofibers [5]. Their fate is regulated by the expression of various transcription factors, including paired box 7 (Pax7) and myogenic differentiation (MyoD) [6]. Pax7 is expressed in quiescent and activated SCs, whereas MyoD is mainly expressed in proliferating and differentiating myoblasts [6, 7].

Recently, research on regenerative medicine has advanced in the field of laryngology. The basic fibroblast growth factor (bFGF) is a member of the fibroblast growth factor family and is a key protein in regenerative medicine. bFGF has already been used clinically and has been shown to be effective in vocal fold sulcus, vocal fold atrophy, and vocal fold scarring [8]. bFGF is known to have potential angiogenic [9] and nerve regeneration effects [10] on muscle tissue and to promote the proliferation of myoblasts [11]. We previously reported that local injection of single, high-dose bFGF into the paralyzed vocal fold 1 month after RLN transection compensated for an atrophied TA muscle by inducing activation of SC proliferation and increasing the number of myoblasts in the RLN paralysis model of rats [12]. Considering the muscle growth effects of bFGF administration after muscle atrophy and the nerve regeneration effects of bFGF [10, 13–15], we speculated that bFGF might have a suppressive effect on muscle atrophy after nerve transection and may accelerate nerve regeneration. Thus, early administration of bFGF before TA muscle atrophy after RLN resection might be more effective in improving the prognosis of vocal function. In addition, if the RLN is severed during surgery or trauma, treatment would be preferable earlier. However, the optimal time of bFGF administration is unknown, and the effects of bFGF administration early after RLN transection on the paralyzed TA muscle have not been examined.

Therefore, this study aimed to elucidate the effects of early bFGF administration on the TA muscle after RLN transection. Furthermore, we investigated the background mechanisms, focusing particularly on the activation of muscle SCs and the nerve regeneration effects of bFGF.

## Methods

### Animals

Ten-week-old male Sprague–Dawley rats weighing 330–370 g were purchased from CLEA Japan, Inc. (Tokyo, Japan) and housed in a temperature-controlled environment under a 12-h light–dark cycle with access to food and water ad libitum [10]. All animal experiments were conducted in accordance with institutional guidelines and with the approval of the Animal Care and Use Committee of the University of Tokyo (No. P16-065).

### RLN paralysis model

The RLN paralysis model was prepared as described previously [12]. The left RLN was exposed and transected at the level of the seventh tracheal ring under general anesthesia. The distal and proximal ends of the nerve were ligated using a 4–0 silk suture, and the proximal end was embedded in the sternohyoid muscle to prevent contact between the cut ends.

### Experimental protocol

**Fig. 1:**
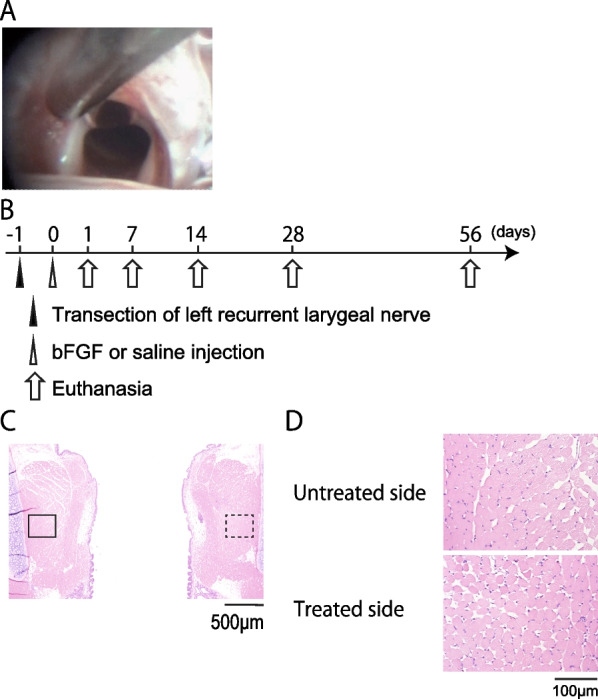
**A** Endoscopic image at injection. Saline or different doses of basic fibroblast growth factor (bFGF) (200 and 2000 ng: low and high dose, respectively) were injected into the left thyroarytenoid (TA) muscle using laryngeal endoscopy under general anesthesia. **B** Experimental timeline. The left recurrent laryngeal nerve (RLN) of rats was transected on day-1. Saline or different doses of bFGF were injected into the left TA muscle transorally on day 0. The laryngeal tissues were collected on the indicated days. **C**, **D** Representative images of hematoxylin and eosin-stained sections of the larynx 1 day after left RLN transection (**C**, 40 × magnification; **D**, 400 × magnification). The solid and dotted rectangles in (**C**) indicate the TA region of the muscle of the untreated and treated sides, which are shown at a higher magnification in (**D**)

Rats were allocated to the following three treatment groups (n = 6 each): (1) saline (saline group), (2) low-dose bFGF (low-dose group), and 3) high-dose bFGF groups (high-dose group). First, the left RLN was cut in all animals under general anesthesia (Day-1). The following day, 10 μL saline, 200 ng bFGF (low-dose) (Fiblast; Kaken Pharmaceutical Company, Ltd., Tokyo, Japan), and 2000 ng bFGF (high-dose) in 10 μL saline were injected into the left TA muscle using laryngeal endoscopy under general anesthesia in each group (Fig. 1A). The bFGF dose was determined based on a previous study [12].

### Tissue preparation

Laryngeal tissues were harvested for histological analysis on days 1, 7, 14, 28, and 56 after saline or bFGF injections (Fig. 1B). The rats were deeply anesthetized and euthanized by sequential intracardiac perfusion with 20 mL saline and 10% formaldehyde. Immediately after euthanasia, the larynx was gently irrigated with 10% formaldehyde to minimize mechanical damage to the laryngeal mucosa and submucosal tissue. After excision, tissue samples were fixed in 10% formaldehyde for 24 h, decalcified for 7 days using decalcifying solution B (Wako Pure Chemical Industries, Ltd, Osaka, Japan), dehydrated in a series of graded ethanol solutions, and then embedded in paraffin.

### Histological analyses of TA muscle

Following the previously reported method [12], histological analysis was performed on the coronal sections of the larynx to evaluate the whole TA muscle and the size of the individual fibers. Images of the bilateral laryngeal area were captured using a digital microscope camera (BZ-X710, Keyence, Osaka, Japan) at 40 × magnification to evaluate the TA muscle (Fig. 1C) and 400 × magnification to examine the individual fiber size (Fig. 1D). We measured the area of the entire TA muscle and individual muscle fibers from microscopic images using ImageJ 1.5108 software (National Institutes of Health [NIH], Bethesda, Maryland, USA) [16]. The evaluator was blinded to the animal grouping and euthanasia time. To calculate the area of the entire TA muscle, the outer circumference of the TA muscle was traced, areas similar in color to the muscle fibers were extracted from its interior, and their areas were summed. To evaluate the area of individual muscle fibers, 50 muscle fibers around the center of the TA muscle were selected, and the average of all areas was calculated. Histological changes in the TA muscle were evaluated by comparing the areas of the treated (T: left) and untreated (U: right) sides using the [T/U] ratio in the same section.

### Immunohistochemistry

The primary and secondary antibodies used in this study are listed in Table 1. Anti-Pax7 and MyoD antibodies were used to assess the changes in muscle SC activation after saline or bFGF injection. Ki-67 was used as the cellular marker of proliferation. To evaluate the expression of Ki-67 and Pax7, double immunofluorescence staining was performed using primary antibodies for Ki-67 and Pax7. To investigate the nerve regeneration effects of bFGF, it was necessary to assess the regulation of the neuromuscular junction (NMJ) of the TA muscle, including the expression status of nerve terminals and acetylcholine receptors. An anti-synaptophysin antibody was used to visualize motor axon terminals, and an anti-cholinergic receptor nicotinic beta 1 subunit (CHRNB1) antibody was used to detect acetylcholine receptors. To evaluate the NMJ, double immunofluorescence staining was performed for the laryngeal tissues after saline or bFGF injections using primary antibodies for synaptophysin and CHRNB1. For immunostaining, antigen retrieval was performed using an antigen retrieval solution (S1700, Agilent Technologies, Tokyo, Japan). The sections were then incubated with Blocking One solution (Nacalai Tesque, Tokyo, Japan) for 30 min at room temperature to block nonspecific antibody binding, followed by incubation with primary antibodies at 4 °C overnight and then with secondary antibodies for 1 h at room temperature. The Vector TrueVIEW Autofluorescence Quenching Kit with 4ʹ,6-diamidino-2-phenylindole dihydrochloride (DAPI) (Vector Laboratories, Burlingame, CA, USA) was used to quench autofluorescence and tissue mounting. DAPI was added for nuclear staining.

**Table 1:** The primary and secondary antibodies used in this study

Antibody	Source	Catalog No	Host	Type	Dilution
*Primary antibody*					
Ki-67	Novus Biologicals(Centennial, CO, USA)	NB600-1252	Rabbit	Monoclonal	1:300
Pax7	Santa Cruz Biotechnology(Santa Cruz, CA, USA)	sc-81975	Mouse	Monoclonal	1:300
MyoD	Dako (Tokyo, Japan)	M3512	Mouse	Monoclonal	1:300
synaptophisin	Gene Tex (Irvine, CA, USA)	GTX633821	Mouse	Monoclonal	1:200
CHRNB1	Thermo Fisher Scientific (Waltham, MA, USA)	PA5-76704	Rabbit	Polyclonal	1:200
*Secondary antibody*					
Anti-rabbit IgG (Alexa Fluor® 488)	Abcam (Cambridge, MA, USA)	ab150077	Goat	Polycolonal	1:500
Anti-mouse IgG (Alexa Fluor® 568)	Abcam (Cambridge, MA, USA)	ab175473	Goat	Polycolonal	1:500

As described previously [12], three different microscopic fields (dorsal, middle, and ventral) of each left paralyzed TA muscle cross-section and right non-paralyzed TA muscle cross-section as a normal control were captured at 400 × magnification to assess the expression of Ki-67, Pax7, and MyoD. Sixty muscle fibers in each microscopic field were counted and summed for three fields (180 fibers) in each animal. The percentages of Ki-67, Pax7, and MyoD immunoreactive cells for all counted muscle fibers were examined, and the results were averaged. To assess the NMJ, we counted the number of co-expressed structures of synaptophysin^+^ nerve terminals and CHRNB1^+^ acetylcholine receptors in the entire TA muscle from both the paralyzed and non-paralyzed sides. Changes in the NMJ of the TA muscle were evaluated by comparing the number of treated (T: left) and untreated (U: right) sides using the [T/U] ratio in the same section.

### Statistical analysis

For histological analyses of the TA muscle and immunohistochemical analyses of Ki-67, Pax7, and MyoD, statistical comparisons between groups were performed using Tukey’s multiple comparison test. In addition, for immunohistochemical analyses of synaptophysin and CHRNB1, statistical comparisons between the groups were performed using Tukey’s multiple comparison test. Statistical comparisons were performed using GraphPad Prism software. Moreover, statistical significance was set at *P* < 0.05.

## Results

### High-dose bFGF prevents atrophic changes of TA muscles after RLN transection

**Fig. 2:**
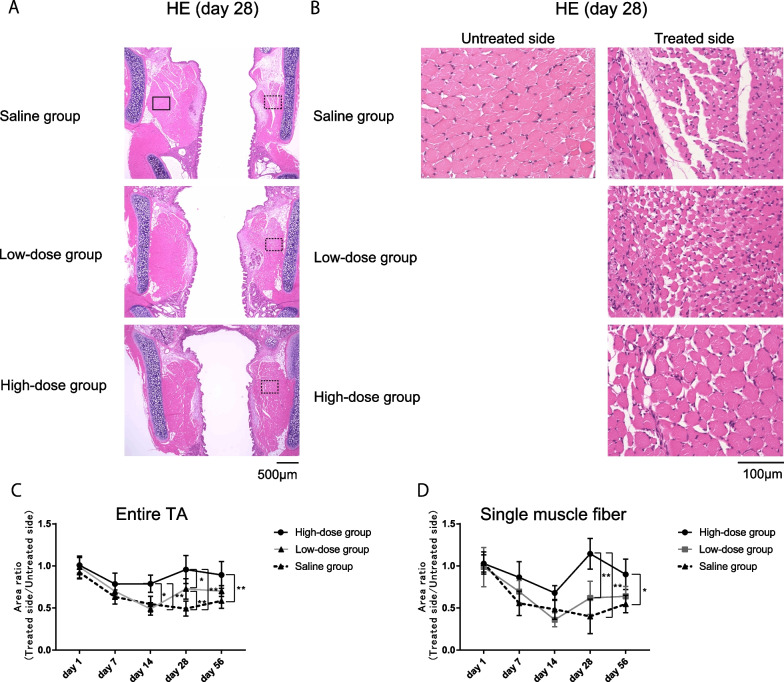
**A**, **B** Representative microscopy images of coronal hematoxylin and eosin–stained thyroarytenoid (TA) muscle sections on day 28 after administration of different doses of basic fibroblast growth factor (bFGF) (200 and 2000 ng: low and high dose, respectively) or saline injection (**A** and **B**: 40 × and 400 × magnification, respectively). The treated/untreated (T/U) area ratio of the entire TA muscle (**C**) and the individual fibers (**D**) of the TA muscle are shown too. The T/U ratio of the entire TA muscle in the high-dose group was significantly larger than that in the saline group on days 14, 28, and 56. The T/U ratio of individual muscle fibers in the high-dose group was significantly larger than that in the saline group on days 28 and 56. Error bars represent standard deviation. *P < .05 and **P < .01, Tukey’s multiple comparisons test

We examined the effects of bFGF on the cross-sectional area of the TA muscles after RLN transection. While we detected a reduction in the T/U ratio of both the entire TA muscle and individual muscle fiber size in the saline and low-dose groups over time, the reduction rate was slight in the high-dose group. The average T/U ratios of the entire TA muscle and individual muscle fibers in the high-dose group on days 28 and 56 were over 0.9 and were significantly greater than those of the saline and low-dose groups (Fig. 2A–D). There was no significant difference between the low-dose and saline groups, except for the entire area of the TA on day 28, in which the low-dose group showed higher values. These results indicate that low-dose bFGF effects were limited, and high-dose bFGF effects were very high, although both high- and low-dose bFGF prevented atrophic changes in the TA muscle after RLN transection.

### High-dose bFGF promoted proliferation of Pax7^+^ SCs of TA muscles

**Fig. 3:**
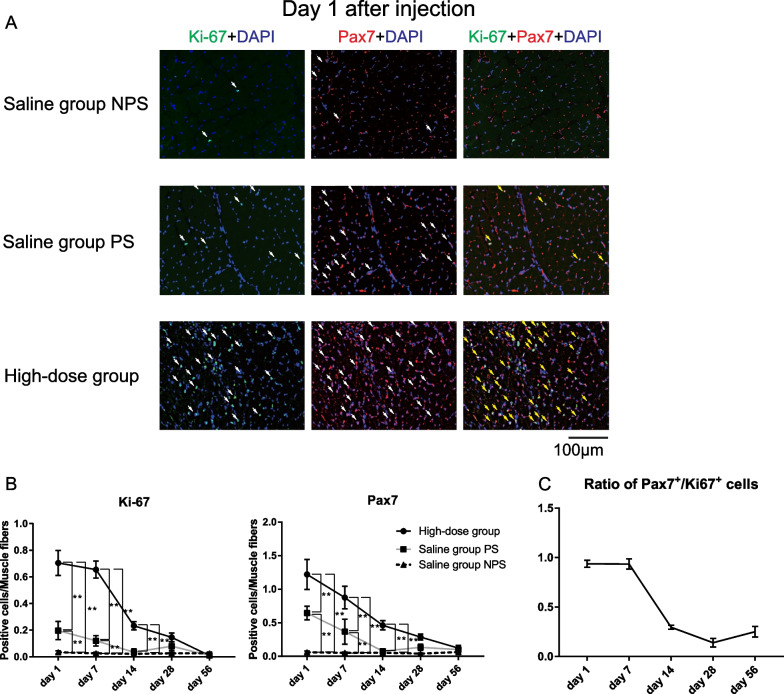
**A** Representative microscopy images of triple staining with Ki67, paired box 7 (Pax7), and 4ʹ,6-diamidino-2-phenylindole dihydrochloride (DAPI) on day 1 after basic fibroblast growth factor (bFGF) (2000 ng: high dose) injection in the paralyzed side, or saline injection in the paralyzed side or non-paralyzed side (400 × magnification). The number of Ki67^+^ proliferating cells and Pax7^+^ SCs in the paralyzed side of the high-dose group was higher than that in the non-paralyzed and paralyzed side of the saline group. Moreover, most Ki67^+^ proliferating cells were co-expressed with Pax7 in the high-dose group. White arrows indicate Ki67^+^ or Pax7^+^ cells. Yellow arrows indicate Ki67^+^ /Pax7^+^ cells. **B** Temporal changes in the ratio of Ki67^+^ and Pax7^+^ cells to muscle fibers after bFGF injection in the paralyzed side or saline injection in the paralyzed or non-paralyzed side. Error bars represent the standard deviation. **P < .01, Tukey’s multiple comparisons tests. **C** Temporal changes in the ratio of Pax7^+^/Ki67^+^ cells in all Ki67^+^ proliferating cells in the high-dose group. The ratios were almost 1 on days 1 and 7, indicating that most Ki67^+^ proliferating cells were Pax7^+^ SCs. NPS, non-paralyzed side of the saline group; PS, paralyzed side of the saline group; High-dose group, paralyzed side of the high-dose basic fibroblast growth factor group

To clarify the mechanisms by which high-dose bFGF suppressed atrophic changes in the TA muscles, we investigated its effects on the cell lineage of SCs to muscle fibers. We examined cell proliferation in the TA muscles and performed time-course analyses of the numbers of Pax7^+^ SCs and Ki67^+^ proliferating cells per muscle fiber. We evaluated the paralyzed side of the high-dose group, paralyzed side of the saline group, and the non-paralyzed side of the saline group as normal control. On the non-paralyzed side of the saline group, the number of Pax7^+^ SCs and Ki67^+^ proliferating cells was very low and did not change over time (Fig. 3A). On the paralyzed side of the saline group, the number of Pax7^+^ SCs and Ki67^+^ proliferating cells increased slightly, peaking on day 1 after administration, compared to that on the non-paralyzed side of the saline group, and decreased with time. In the high-dose group, the number of Pax7^+^ SCs and Ki67^+^ proliferating cells increased markedly and significantly, peaking on day 1 after administration, compared with that on both the paralyzed and non-paralyzed sides of the saline group. It decreased with time and was not significantly different on days 28 and 56 compared with the other two groups (Fig. 3A, B).

We examined the ratio of Pax7 + /Ki67 + cells in all Ki67 + proliferating cells in the high-dose group to determine the proportion of Pax7 + SCs in Ki67 + proliferating cells (Fig. 3C). Specifically, most Ki67^+^ proliferating cells were Pax7^+^ SCs, particularly on days 1 and 7. These results suggest that high-dose bFGF administration activates SCs and promotes their proliferation.

### MyoD^+^ myoblasts increased 14 days after high-dose bFGF administration

**Fig. 4:**
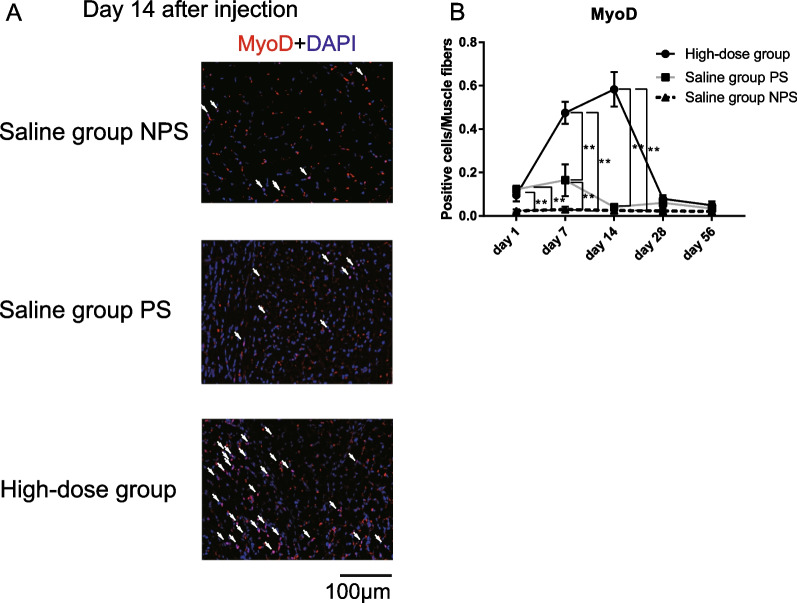
**A** Representative microscopy images of double staining with myogenic differentiation (MyoD) antibody and 4ʹ,6-diamidino-2-phenylindole dihydrochloride (DAPI) on day 14 after high dose basic fibroblast growth factor (bFGF) injection in the paralyzed side, or saline injection in the paralyzed side or non-paralyzed side (400 × magnification). The number of MyoD^+^ myoblasts in the paralyzed side of the high-dose group was higher than that in the non-paralyzed and paralyzed side of the saline group. White arrows indicate MyoD^+^ cells. **B** Temporal changes in the ratio of MyoD^+^ myoblasts to muscle fibers after bFGF injection in the paralyzed side or saline injection in the paralyzed or non-paralyzed side. Error bars represent standard deviation. **P < .01, Tukey’s multiple comparisons tests. Saline group NPS, non-paralyzed side of the saline group; Saline group PS, paralyzed side of the saline group; High-dose group, paralyzed side of the high-dose basic fibroblast growth factor group

To examine the effects of high-dose bFGF on cell differentiation of SCs to myoblasts, we compared the number of MyoD^+^ myoblasts per muscle fiber among the paralyzed side of the high-dose group, the paralyzed side of the saline group and the non-paralyzed side of the saline group as normal control (Fig. 4A). On the non-paralyzed side of the saline group, the number of MyoD^+^ myoblasts did not change over time. In the paralyzed side of the saline group, the ratio of MyoD^+^ myoblasts increased slightly compared to that of the non-paralyzed side of the saline group, with significant differences on days 1 and 7. In contrast, the ratio in the paralyzed side of the high-dose bFGF group was significantly and markedly increased compared to that in the paralyzed and non-paralyzed sides of the saline group on days 7 and 14. The ratio of MyoD^+^ myoblasts increased until day 14, and the peak MyoD^+^ myoblast ratio followed the trend of Pax7^+^ cells, which was observed on day 1 after high-dose bFGF administration (Fig. 4B). These results suggest that high-dose bFGF induces the differentiation of SCs into myoblasts, thereby increasing the TA muscle area.

### NMJs in the paralyzed side of the TA muscles increased 28 and 56 days after high-dose bFGF administration

**Fig. 5:**
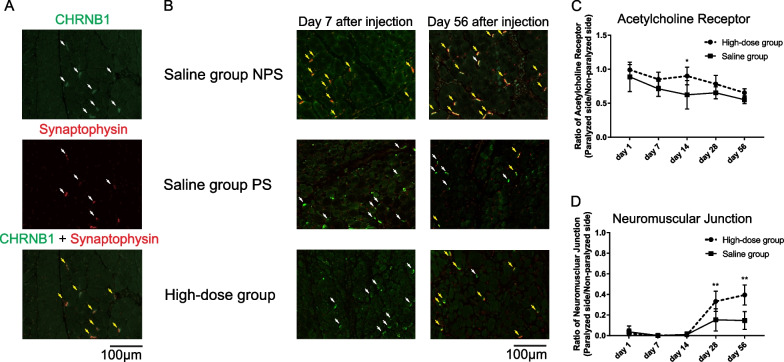
**A** Representative microscopy images of double staining with cholinergic receptor nicotinic beta 1 subunit (CHRNB1) and synaptophysin in the untreated side (400 × magnification). White arrows indicate CHRNB1^+^ acetylcholine receptor or synaptophysin^+^, and yellow arrows indicate CHRNB1^+^/synaptophysin^+^ neuromuscular junction (NMJ). **B** Representative microscopy images of the NMJ on days 7 and 56 after high-dose basic fibroblast growth factor (bFGF) injection in the paralyzed side or saline injection in the paralyzed or non-paralyzed side (400 × magnification). White arrows indicate CHRNB1^+^ acetylcholine receptor, and yellow arrows indicate CHRNB1^+^/synaptophysin^+^ NMJ. **C** Temporal changes in the paralyzed/non-paralyzed ratio of the acetylcholine receptor after bFGF or saline injection in the paralyzed side. **D** Temporal changes in paralyzed/non-paralyzed ratio of NMJ after bFGF injection in the paralyzed side or saline injection in the paralyzed side. Error bars represent standard deviation. *P < .05 and **P < .01, Tukey’s multiple comparisons test. Saline group NPS, non-paralyzed side of the saline group; Saline group PS, paralyzed side of the saline group; High-dose group, paralyzed side of the high-dose basic fibroblast growth factor group

Finally, we examined the nerve regeneration effects of bFGF and evaluated the number of NMJs in the paralyzed TA muscle (Fig. 5). Acetylcholine receptors were labeled for CHRNB1 immunoreactivity, and NMJs were co-labeled for CHRNB1 and synaptophysin immunoreactivity (Fig. 5A). Acetylcholine receptors decreased gradually with time in both the high-dose and saline groups. Although acetylcholine receptor levels were slightly higher in the high-dose group, there was no significant difference, except on day 14 (Fig. 5C). On the paralyzed side, NMJs were almost absent until day 14 in both the high-dose and saline groups. However, on days 28 and 56, the NMJs became scattered. In both days 28 and 56, NMJs were significantly higher in the high-dose group, with an average T/U ratio of 0.15 and 0.12 in the saline group, respectively, and 0.33 and 0.39 in the bFGF group, respectively (Fig. 5B, D). These results suggest that high-dose bFGF promotes reinnervation in the paralyzed side of the TA muscle.

## Discussion

The current study has demonstrated that high dose bFGF early after RLN transection significantly increases Ki67^+^ proliferating cells and Pax7^+^ SCs, followed by MyoD^+^ myoblasts, and then NMJs and thereby prevents atrophic changes in TA muscle.

SCs are precursors of skeletal muscle cells and are normally in a quiescent state characterized by the absence of cell cycling in preparation for muscle damage, such as denervation or injury. Pax7 is a transcription factor essential for maintaining normal function and is characteristically expressed in quiescent or activated muscle SCs [17]. When muscle denervation or injury occurs, signals such as bFGF and hepatocyte growth factor are released [18], activating SCs and leading them to enter the cell cycle. Subsequently, the SCs proliferate and differentiate into myoblasts expressing MyoD. MyoD is an important transcription factor that regulates essential proteins such as myosin heavy and light chains, and acetylcholine receptor [19]. Myoblasts migrate and proliferate toward myofibers for repair.

In the present study, Pax7^+^ SCs of the TA muscles increased slightly in the saline group, with a peak on day 1 after administration, and MyoD-positive myoblasts increased on days 1–7, suggesting that these increases were caused by denervation or stimulation due to myofiber damage from the injection needle. Nonetheless, these increases in Pax7 and MyoD may not be sufficient to maintain the myofiber area since TA atrophy progresses with time. bFGF has been shown to activate SCs in vitro [11], and the high-dose group, exogenous bFGF resulted in a marked increase in Pax7^+^ SCs and MyoD^+^ myoblasts compared with the saline group. However, the significant difference in Pax7^+^ SCs and MyoD^+^ myoblasts between the saline and high-dose groups disappeared by day 28, indicating that SC activation by bFGF infusion is only a short-term effect.

Regarding the timing of administration, our previous study [12] showed that when high-dose bFGF was administered 1 month after paralysis, the mean peak numbers of Ki67, Pax7, and MyoD per muscle fiber were 0.2, 0.4, and 0.3, respectively, which were lower than those obtained with early injection after paralysis in this study. Regarding SC activation, it is likely to be higher when treated in the early phase after paralysis.

Detailed studies of whole human larynges obtained from autopsies have shown that, in many cases, the RLN anastomoses with the internal or external branches of the SLN inside the larynx [4, 20]. In general, when nerve-innervating muscle fibers are degenerated, sprouting of adjacent normal nerve fibers reforms NMJs, targeting the remaining acetylcholine receptors [21, 22]. Similarly, when the RLN is paralyzed, it has been reported that nerve reinnervation by the SLN or autonomic nerve may occur within the denervated TA muscle [23]. Once muscle fibers are reinnervated, acetylcholine and neurotrophic factors are thought to inhibit and ameliorate the atrophy of denervated myofibers [24, 25]. In the saline group in this study, a small number of NMJs were observed in the muscle fibers at 28 and 56 days after paralysis, suggesting that these muscle fibers were reinnervated by some normal nerve fibers. Exogenous bFGF can regenerate Schwann cells [13–15], axons [14, 15], and acetylcholine receptors [14]. bFGF administration may have increased the number of NMJs in the high-dose group by enhancing neuronal reinnervation effects. Taken together, the effects of bFGF were thought to promote muscle regeneration by activating SCs and myoblasts up to 28 days after administration and to maintain muscle fibers by reforming NMJs in the mid to long term.

This study had several limitations. First, although high-dose bFGF significantly increased the number of NMJs, the increase was only approximately 40% relative to the untreated side, so it is unclear whether the effect of maintaining TA muscle volume lasts longer. However, even though the SC activity decreased and was no longer significantly different between the high-dose and saline groups, atrophy of the TA muscle was suppressed 56 days after administration, suggesting that reinnervation may be sufficient to maintain the TA muscle. Once denervated muscle fibers are reinnervated, their innervation is considered permanent, and it is inferred that atrophy is suppressed even in the long term. Second, although the number of NMJs increased after high-dose bFGF administration, we did not know whether the nerve of origin was the superior laryngeal nerve, autonomic nerve, or another nerve and whether it produced muscle activity during speech. Electromyography of vocal fold muscles needs to be investigated, but this is a topic for future study.

## Conclusions

This study examined whether early local administration of bFGF to the TA muscle after recurrent nerve transection can prevent atrophy of the TA muscle. Local injection of high-dose bFGF was shown to activate SC proliferation, increase myoblasts, and reform NMJs, which disappeared after transection of the recurrent nerve. These results suggest that a single injection of high-dose bFGF early after paralysis may prevent atrophy of the TA muscle in the mid to long term.

## Data Availability

The datasets used and/or analyzed during the current study are available from the corresponding author on reasonable request.

## Abbreviations

UVFP: Unilateral vocal fold paralysis
RLN: Recurrent laryngeal nerve
TA: Thyroarytenoid
SLN: Superior laryngeal nerve
SC: Satellite cell
Pax7: Paired box 7
MyoD: Myogenic differentiation
bFGF: Basic fibroblast growth factor
NMJ: Neuromuscular junction
CHRNB1: Anti-cholinergic receptor nicotinic beta 1 subunit
DAPI: 4ʹ,6-Diamidino-2-phenylindole
